# Clonal redemption of B cells in cancer

**DOI:** 10.3389/fimmu.2023.1277597

**Published:** 2023-10-26

**Authors:** Tyler R. McCaw, Serena Y. Lofftus, Joseph G. Crompton

**Affiliations:** Department of Surgery, Division of Surgical Oncology, University of California, Los Angeles, CA, United States

**Keywords:** clonal redemption, cancer, atypical memory B cell, polyreactive antibodies, anergic B cells, autoimmunity

## Abstract

Potentially self-reactive B cells constitute a large portion of the peripheral B cell repertoire in both mice and humans. Maintenance of autoreactive B cell populations could conceivably be detrimental to the host but their conservation throughout evolution suggests performance of a critical and beneficial immune function. We discuss herein how the process of clonal redemption may provide insight to preservation of an autoreactive B cell pool in the context of infection and autoimmunity. Clonal redemption refers to additional recombination or hypermutation events decreasing affinity for self-antigen, while increasing affinity for foreign antigens. We then review findings in murine models and human patients to consider whether clonal redemption may be able to provide tumor antigen-specific B cells and how this may or may not predispose patients to autoimmunity.

## Introduction to clonal redemption

A complex series of recombination events during development inexorably culminates in formation of self-reactive B cell receptors. Although these cells can be eliminated prior to egress from the bone marrow in central tolerance, a significant number of self-reactive B cells can be isolated from the peripheral blood of healthy individuals; indeed, 55-75% of new immature B cells and 20% of mature naïve B cells in humans are potentially autoreactive (1). These cells can escape peripheral deletion through adopting a state of anergy or hyporesponsiveness. Persistence of these autoreactive cells, though, begs the question: what is the purpose of maintaining such a significant autoreactive B cell population? Evolutionarily, this is ostensibly counterproductive as it represents a substantial energy expenditure as well as possible autoimmune pathology, detrimental to the host. Conversely, elimination of all self-reactive B cells is anticipated to create large gaps in the B cell repertoire that could be exploited by pathogens (2). Maintenance of a low-affinity polyreactive pool, capable of recognizing self or pathogenic antigens, can serve as a compromise, providing an early or first-line response to rapidly address a pathogenic threat (3). Then, to mitigate long term autoimmune effects, these autoreactive cells can enter a germinal center (GC) where they might undergo somatic hypermutation (SHM) with selection of mutants that are specific for antigen and deletion of more autoreactive mutants. This was demonstrated in murine B cells specific for HEL antigen, wherein an initial S52N mutation in CDR2 decreases affinity for self-antigen, followed by accrual of subsequent mutations away from autoreactivity to enable self versus non-self discrimination (4). This phenomenon, termed “clonal redemption”, describes entry of self-reactive B cell clones into the GC wherein SHM abrogates autoreactivity and promotes increased affinity for a specific antigen. Direct experimental evidence for clonal redemption in humans was provided by analysis of three antibodies with autoreactive preimmune sequences. The authors focused on heavy chain V segment IGHV4-34*01, which contains a hydrophobic patch conferring autoreactivity to the red blood cell antigen poly-N-acetyl-lactosamine and agglutination thereof. SHM disrupted the hydrophobic patch to abrogate binding to self-antigen and increased affinity for RhD or vaccinia. Notably, mutations decreasing affinity for self- and increasing affinity for foreign antigen were non-overlapping, suggesting distinct mechanisms governing both processes (5).

In the context of cancer—since many cancer antigens are self or near-self—a theory of clonal redemption may provide a useful conceptual framework to investigate the relationship between B cell-mediated anti-tumor immunity and B cell–mediated autoimmunity in cancer patients. In this review, we expand the definition of clonal redemption to include elaboration of tumor-reactive antibodies via entry into the GC or extrafollicular production without further SHM. The overall goal is to develop an understanding that would result in therapeutic approaches that minimize B cell-driven autoimmune events while concomitantly enhancing the therapeutic efficacy of humoral anti-tumor immunity.

## Humoral immunity in human cancer

Humoral immunity mediated by B cells is critical in response to both acute and chronic infections. Recent evidence also points to a role for B cells in anti-tumor immunity, as presence of B cells and tertiary lymphatic structures (TLS) in the tumor microenvironment (TME) correspond with improved patient outcomes in a variety of cancers including: high grade serous ovarian (HGSOC) (6), colorectal (7), gastric (8), melanoma (9), sarcoma (10), tongue squamous cell carcinoma (11), cervical squamous cell carcinoma (12), and lung (13). Particularly, in breast cancer increased frequencies of TIL-B correlate with an increased T cell infiltrate as well (14, 15). In node positive HER2+ and TNBC patients, increased TIL B further demonstrated a positive correlation with increased TLS as well as disease free survival and overall survival (15).

An inherent autoreactive proclivity is present within the B cell compartment as evidenced by the ability to produce antibodies to tumor antigens, which often possess highly concordant structures with native protiens. Here, potential tumor antigens, or near-self antigens, include bonafide neoantigens (secondary to somatic mutations), overexpression of native proteins, ectopic protein expression, altered post-translational modifications, or potentially alterations of protein structure within the TME. As tumors arise from autologous cells expressing self-antigen, the production of antibodies to self- or near-self antigens has been thought to represent improved immune surveillance in the setting of increased presentation of tumor self-antigen, and autoantibodies have been suggested as a prognostic biomarker for early disease in a number of cancer types (16–19). (For a comprehensive review on tumor-associated antibodies see Laumont et al. (20)).

## Atypical B cell as candidate clonal redemption population in humans

Human atypical B cells (ABCs) were initially defined as CD21- CD27- (21) and functionally hyporesponsive to chronic antigen exposure in malaria and HIV. This was also demonstrated following *in vitro* stimulation with BCR ligation, CD40, TLR9, showing reduced calcium flux and proliferation (22). Moreover, these cells undergo CSR and SHM but to a lesser extent than their classical memory CD27+ counterparts (21, 23). This suggests they are antigen experienced and may adopt a long-lived, memory program analogous to exhaustion. Study of ABCs has been confounded by inconsistent definitions used in the literature; however, both mouse and human ABCs appear to express CD11c, Tbet, Zeb2, FCRL5 among others (24–26), redemonstrated by next generation RNA-sequencing analysis (24, 27). FCRL5+ ABCs are formed in both acute and chronic infections, though their frequency is significantly increased following chronic antigen exposure (28). These cells do not express CXCR5, CCR7, and CD62L but are positive for CCR6 and CXCR3, which likely explains their preferential localization to non-lymphatic tissues and sites of inflammation (21–23).

Early observations revealed that Tbet and STAT1 expression in circulating CD19+ B cells of patients with SLE are significantly higher than healthy controls (29). Indeed, IL-21 can potently induce CD11c^hi^ Tbet+ B cells from SLE patients and promote differentiation into autoreactive antibody secreting cells (ASCs). This then provides context to the observed correlation between ABCs, defined as CD11c^hi^, and reactivity to many self-antigens (55 of 95 autoantigens, including dsDNA, nucleosome, histones, RNP, Smith, La, chromatin) as well as a disease severity score (30). These human studies in SLE are thereby concordant with murine studies demonstrating that Tbet+ B cells are a multipotent memory population with ability to generate autoantibody producing cells.

A similar ABC population is found in malarial infection but does not appear to be pathologic in this context. Indeed, antibodies from ABCs in patients with malaria possess specificity to Plasmodium falciparum as well as autoantigens (31, 32). Patients with active *Plasmodium vivax* infection were found to produce more Tbet^hi^ atypical memory B cells compared to non-exposed individuals, which had switched IgG, increased expression of FcRL5, and reduced Syk phosphorylation on BCR ligation. These were maintained for at least 3 months post infection. Here, IFNγ, TLR7/8, and/or IL-21 were required for differentiation into ASCs (33). This is consistent with an antigen experienced, switched ABC population that is hyporesponsive to BCR signaling in malaria. Reasons for observed discrepancies between malarial infection and SLE remain elusive but may relate to failure in regulatory mechanisms. For example, B cells in patients with SLE have incomplete X inactivation by the lncRNA XIST, yielding increased dosage of TLR7 and genes related to IFNγ production, poising cells for ABC differentiation and autoimmunity (34). Additionally, while ABCs represent a distinct differentiation fate that is largely conserved across disease states, transcriptional analysis revealed heterogeneity in this subset that might contribute to pathology-specific outcomes (27).

## Atypical B cells in cancer

B cells with overlapping phenotypes as ABCs have recently been reported in several human cancers, suggesting their trafficking to and accumulation within the TME. In 32 patient samples of SCC of the tongue, increased frequencies of CD19+ CD27- IgD- IgM- B cells correlated with reduced disease burden in the lymph nodes (35). In an analysis of patients with high grade serous ovarian cancer, the majority of CD20+ TIL were IgD- IgM- IgG+, indicating antigen experience and class switching, showed evidence of SHM, and demonstrated increased clonality. The majority of these TIL-B were also CD27-, suggesting an ABC phenotype (36). The authors note, however, a paucity of GC B cells and plasmablasts by flow and do not note organized TLS by immunofluorescence, suggesting that this SHM may be occurring in the tumor-draining lymph nodes with trafficking of these clones to the TME. In a study of 120 cases of HCC, infiltration of the tumor margin with CD20+ B cells correlated with improved patient outcomes. These cells tended to have a phenotype consistent with switched ABCs—CD27- CD38-, IgD-, IgM-, IgG+. Moreover, these cells produced IFNγ by flow cytometry, intimating Tbet expression. Ex vivo killing assays demonstrated an ability of these cells to directly kill tumor cells as well as express granzyme B and TRAIL. Although, production of tumor-reactive autoantibodies was not assessed (37). In a cohort of early-stage breast cancer samples, CD21- CD27- IgD- CXCR5- B cells, suggestive of ABCs, were enriched in the TME. Pre-treatment, these represented about 35% of CD27- IgD- B cells, while expanding to nearly 90% post-chemotherapy (38). It is tempting to speculate that chemotherapy-induced liberation of damage-associated molecular patterns may signal through TLRs to participate in ABC stimulation and proliferation. Collectively, these studies demonstrate that antigen experienced, class switched ABCs accumulate within the human TME and seem to correlate with improved outcomes. Upregulation of IFNγ and granzyme B suggest these cells can directly engage and kill tumor cells in the TME. Studies investigating antibody specificity for tumor antigens and propensity to form intratumoral TLS are still needed to understand the role these cells have in tumor-specific antibody production and whether tumor TLS can support SHM of these populations.

## Lessons from murine studies

Studies of autoreactive antibody production in mice have focused on a role for innate signaling through TLRs and IFNγ signaling with consequent induction of the transcription factor Tbet. This line of evidence began with description of CD11c+ CD11b+ or CD21- CD23- B cells in aging mice (25, 39), now known to encompass ABCs. Indeed, Tbet overexpression is sufficient to drive a CD11b+ CD11c+ B cell phenotype. Tbet and CD11c expression in B cells can also be induced by BCR engagement in cooperation with TLR7 or TLR9 and IFNγR signaling. This population is induced following infection with multiple murine viruses and B cell specific Tbet knockout drives significantly increased titers of gamma herpes virus 68 (40). These results suggest ABCs are necessary for control of viral load. Moreover, that Tbet expression is common to ABCs and may be a master regulator of this cellular program.

IgG2 (particularly IgG2a) is the most effective isotype for controlling viral infections in mice (41, 42) and class switch recombination (CSR) to this isotype can be regulated by Tbet in a T cell-independent fashion. This has been demonstrated with enforced expression of Tbet (43) as well as stimulation with LPS and IFNγ (44). BCR and TLR signaling can also cooperatively induce CSR and AID through non-canonical NFκβ pathway in a T cell-independent manner (45). Interestingly, *in vitro* culture of Tbx21-/- B cells with anti-IgM, R848, IFNγ, IL-21, and anti-CD40 can induce a CD11c+ CD11b+ phenotype, likely encompassing ABCs. However, frequencies of CD11c+ CD11b+ B cells were significantly reduced and produced significantly less IgG2c in Tbet deficient cells (46). Together these results suggest that ABCs might be generated without Tbet but that these cells likely do not achieve the same functional capacity as Tbet replete ABCs, or that Tbet is instead required for maintenance of this cellular program.

Importantly, CSR to IgG2a/c is also associated with autoimmunity as these isotypes are enriched for autoreactive antibodies. A role for Tbet+ B cells in autoimmunity then is unsurprising. For example, B cell specific Tbet deletion (under the control of CD19 Cre) significantly reduces kidney pathology and improves overall survival in several murine models of lupus. Development of autoantibodies (anti-chromatin) and CSR to IgG2a is significantly delayed with a concurrent reduction in frequency and number of CD11c+ B cells in these knockouts (47). Tbet expression in B cells can also be induced in an IFNγ independent fashion through TLR-MyD88 signaling (48). It follows that chronic stimulation of WT B6 mice with TLR7 agonist (but not TLR9) led to development of anti-Smith autoantibodies in a MyD88 dependent fashion. This could be rescued with deletion of ABCs in CD11c-DTR mice (25). Using wiskott-aldrich syndrome protein deficient (was-/-) chimeric mice with B cell specific TLR7 deletion similarly yielded reduced development of autoantibodies and progression of lupus-like glomerulonephritis. Interestingly, in this model, deletion of TLR9 exacerbates kidney pathology and generates a broad range of IgG2c autoantibodies (49). This is in contrast to work by Ehlers et al. showing that signaling through TLR9-MyD88 is required for class switching and increased formation of autoreactive IgG2a and IgG2b antibodies in another murine model of lupus (50). Finally, Tbet expression can also be induced *in vitro* with TLR4 signaling in combination with IFNγ or IL-27 (51). Collectively, these results suggest potentially non-redundant signaling through different TLRs in combination with BCR in response to unique environmental milieu for the generation of ABCs.

These studies point to a role for ABCs in direct cellular cytotoxicity as well as humoral immunity and autoimmunity. This range of activities can be explained by the observation that Tbet+ IgM+ B cells–encompassing CD11c+, CD11c-, and interconversions thereof–can differentiate into all effector and memory lineages, while also ensuring long-term persistence through self-renewal (52). Since up to 90% of immature B cells are self-reactive (53), autoreactive Ig predisposing to autoimmunity can potentially be generated through failure of SHM and inability to alter antibodies from autoreactivity (54) or incomplete B cell diversification after entry into the GC (55). Collectively, this data implicates ABCs as a source of autoreactive antibodies in murine models of autoimmunity. It is conceivable that these autoreactive ABCs may serve as a substrate for clonal redemption and generation of tumor-specific antibodies, targeting near-self or overexpressed self-antigens.

## Evidence for clonal redemption of B cells in cancer

From an evolutionary perspective, clonal redemption is postulated to have developed in order to avoid large gaps in the B cell repertoire and better combat infection. In contrast, because cancer typically afflicts organisms after reproductive age it would not be expected to exert a selective pressure for such a potentially dangerous mechanism. This then raises the question: can clonal redemption contribute to an anti-tumor immune response? There is emerging evidence that suggests accumulation of ABCs within the TME. Although it seems likely that ABCs would be able to undergo clonal redemption and generate tumor-reactive antibodies, this has yet to be explicitly demonstrated.

**Figure 1:**
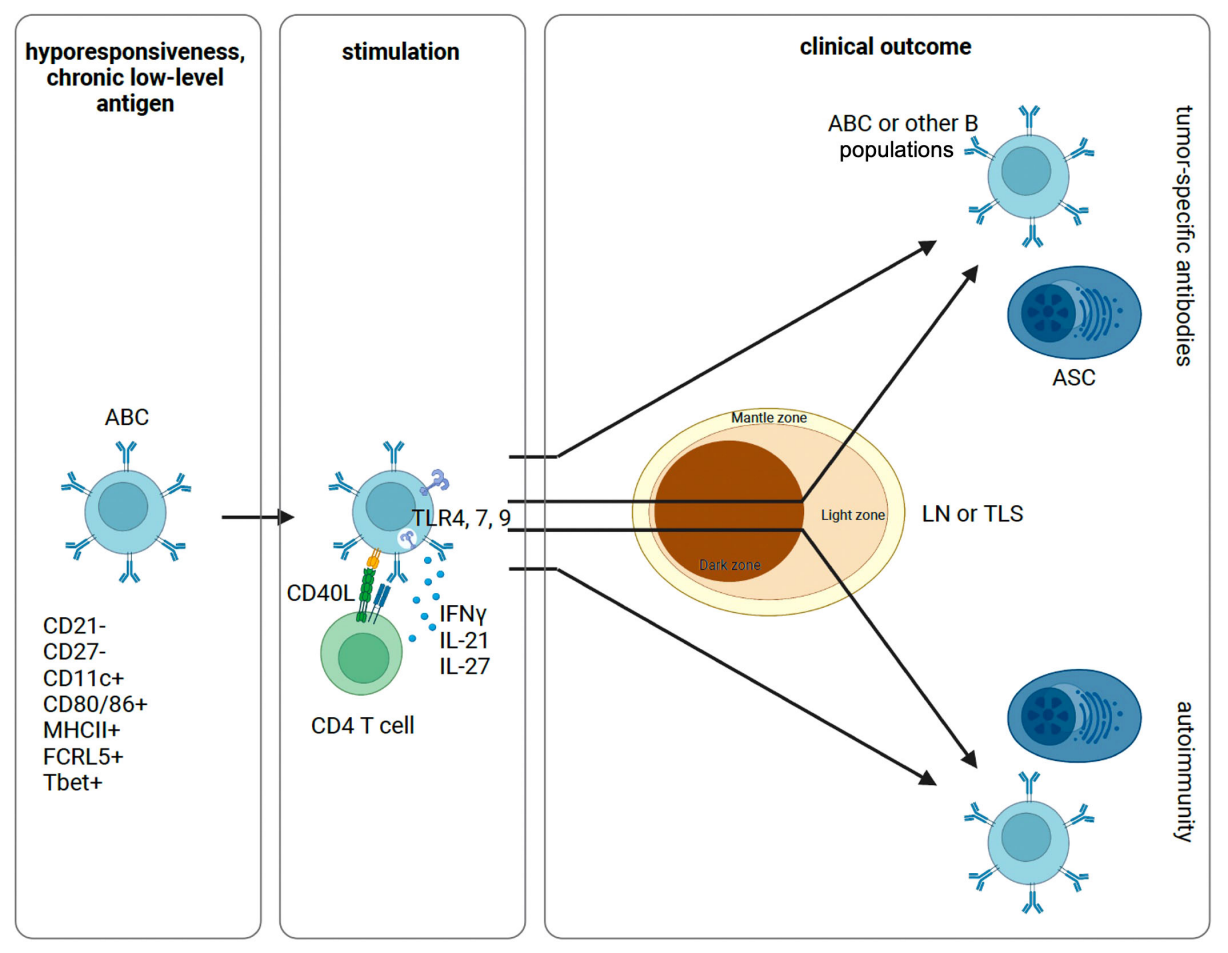
Proposed schematic of ABC activation and differentiation. Following initial antigen encounter, ABCs are generated and maintained in a state of hyporesponsiveness. Sufficiently strong signals through a combination of the BCR, TLRs, and cytokines can stimulate anergic B cells with or without T cell help. These cells can then differentiate into ASCs or other B cell populations. In this process, ABCs can re-enter the GC for additional SHM or not. Depending on the inflammatory context and specific constellation of signals provided, this process can yield tumor-specific antibodies or self-reactive antibodies causing autoimmunity. ABC, atypical memory B cell; ASC, antibody secreting cell; LN, lymph node; TLS, tertiary lymphatic structure.

Recently, Mazor et al. demonstrated IgG binding to the surface of tumor cells from a range of primary human tumor tissue specimens. Their findings suggested a functional relevance to the autoantibodies observed in cancer, as tumor coating was associated with improved patient outcomes. Analysis focused on high grade serous ovarian (HGSOC), and these patients harbored frequent autoantibodies to MMP14 (overexpressed in HGSOC). They next sequenced heavy and light immunoglobulin chains of several intratumoral ASCs and reconstructed clonal lineage trees to show clonal diversification through progressive SHM. Their findings suggest tumor-specific antibodies can be generated secondary to SHM (denoted class I) or from germ-line sequence (denoted class II). After SHM reversion, a similar number of analyzed MMP14-reactive antibodies exhibited decreased binding as no change in binding. Additionally, there were two cases of increased binding after SHM reversion. Interestingly, in a large retrospective analysis there was no increased incidence of autoimmunity of HGSOC patients with autoantibodies (56). This work demonstrates stepwise maturation of IgG sequence toward a defined tumor antigen—overexpression of a native protein—in a manner that apparently does not impose undue predisposition to autoimmunity. Notably, a comparable number of tumor-reactive antibodies were found to arise from germline sequences without further SHM. It should be mentioned that the small number of patient samples used for this analysis (four) may limit the generalization of their findings. Others have similarly observed frequent autoantibodies to over- or ectopically-expressed proteins in the context of tumors, including p53, the cancer testis antigen NY-ESO-1, as well as other intra- and extra-cellular proteins (57, 58). These observations raise the possibility that autoreactive ABCs can be stimulated and differentiate into ASCs through one of two routes: (i) re-entry into the GC for additional SHM and mutation away from self or (ii) extrafollicular production of autoantibodies without further SHM (**Figure 1**). Because many identified tumor antigens are over- or re-expressed native proteins (59, 60), this also provides important evidence that autoantibodies targeting native proteins overexpressed on tumors can be raised without overt autoimmunity.

A major obstacle limiting our understanding of a possible role for clonal redemption in cancer is identification and isolation of a tumor neoantigen-specific BCR. Here, repertoire cloning could facilitate lineage tracing and direct evidence for clonal redemption in cancer, as has been done following infection or vaccination with defined antigens. In murine studies, the presence of a defined antigen would enable transfer of ABCs into tumor-bearing mice with subsequent lineage tracing and mapping of clonally related BCRs. In this way providing direct evidence for clonal redemption as well as the spectrum of potential ABC fates in the context of cancer. Phenotypic and transcriptional analysis of that lineage could then inform possible therapeutic strategies. It is also unclear whether clonal redemption could be accomplished in a mature tumor-associated TLS. Because ABCs do not express typical homing receptors for secondary lymphatic tissues, it is unclear if they might be able to enter a GC, or if clonal redemption might take place through extrafollicular mechanisms. Although it is tempting to speculate, this area of investigation is yet in the early stages and our understanding of whether clonal redemption plays a biologically significant role in cancer will continue to evolve.

## CD5 expression marks another tumor-reactive B cell population

Tumor reactive antibodies may also derive from an innate-like population of B cells characterized by CD5 expression (also called B-1a cells) in humans and their Ly-1 expressing counterparts in mice. Although expanded in disease states, these populations are also a normal component of the circulating B cell repertoire in healthy subjects (61), accounting for approximately 20% of CD20+ cells (62, 63). A distinctive feature of these cells is their propensity to produce polyreactive antibodies—targeting self and foreign antigens (64). These “natural autoantibodies” are those derived from germline sequences, typically IgM, and characterized by low affinity (K_d_ of approximately 10^-4^ to 10^-6^) polyreactive binding (65), though CD5+ B cells can also produce monoreactive, high affinity antibodies suggestive of antigen-driven clonal selection (66). Antibody targets are diverse and include self (proteins, carbohydrates, lipids, nucleic acids) and non-self (virus, bacteria) antigens (67). Their broad binding potential is due to a flat, flexible binding pocket capable of adopting various configurations to accommodate different antigens (68). Such structural promiscuity is conferred by skewed use of VH sequences (69, 70) and frequent use of the VH4-34 sequence (71). VH4-34 encoded antibodies are inherently autoreactive recognizing straight chain poly-N-acetyl-lactosamine on RBCs and B cells (72).

Abundance in the neonatal period and waning frequencies with age (though consistent numbers) suggest a phylogenetically distinct role for this population. Use of germline sequences and T cell independent antibody secretion enables these innate-like cells to sequester commensal bacteria within the gut and provide a first line of defense against pathogens (73, 74). To prevent overt autoimmunity, autoreactive B cells are typically excluded from the GC preventing IgG CSR and memory formation; although, this tolerance mechanism is compromised in the context of SLE (75, 76).

The above properties may also confer CD5+ B cells with anti-tumor potential. Because CD5+ B cells, particularly those utilizing VH4-34, often bind carbohydrate antigens, these populations may be able to recognize the aberrant glycosylation and post-translational modification patterns in tumors (77). In fact, the ability of natural antibodies produced by CD5+ B cells to recognize cancers of the stomach, colon, pancreas, esophagus, lung, prostate, breast, and skin (melanoma) through binding to carbohydrate moieties has been demonstrated (78). Remarkably, these antibodies bind to precancerous and cancerous tissues but not to normal adjacent tissue (78–81). Antibody binding to tumor cells was able to induce apoptosis during *in vitro* culture (78, 80). Although breakdown of tolerance mechanisms in a tumor draining lymph node and entry into the GC, as seen in SLE, could provide an avenue for clonal redemption, this is unlikely to be the case as the tumor-reactive antibodies from the above studies were predominantly IgM, had little evidence of SHM, and increased mutations actually resulted in decreased tumor binding. Thus, CD5+ B cells might recognize tumors through increased avidity of IgM antibodies for overexpressed proteins or aberrant glycosylation patterns leading to structural differences in tumor proteins.

Defining features of ABCs and CD5+ B cells intuitively invite comparison. Both are poised for antibody production without T cell help, are able to produce autoantibodies, and can express high levels of costimulatory molecules (82). Both are subject to a core cellular program of anergy due to chronic recognition of self-antigen, and CD5 is a negative regulator of BCR signaling to help enforce anergy (83). Furthering this point, single cell RNA-sequencing of murine CD5+ and CD21^low^, CD23^low^ B cells (taken to represent ABCs in this study) revealed anergy in both populations to be established by EGR2/3, whose expression was proportionate to downregulation of surface IgM by self-antigens. Importantly, though, these populations largely segregated on clustering analysis (84). Therefore, while these populations share many overlapping features, their potential interrelatedness has been difficult to discern. One issue has been inconsistent definitions of ABCs in the literature frequently obfuscate direct comparison. Moreover, B-1 cells can either express CD5 at the protein level (B-1a) or only the mRNA level (B-1b) (85); therefore, studies of CD5+ cells by immunophenotyping may not capture the B-1b population. Reconciliation of several key differences may help resolve the issue. First, Tbet, a transcription factor induced by IFNγ and responsible for its transcription (86), is central to the ABC program; however, IFNγ signaling disrupts the CD5+ program when IL-10 is depleted (87). Another notable discrepancy is the capacity for transdifferentiation. Transfer experiments in mice have demonstrated that ABCs can give rise to all B cell lineages, while transfer of B-1 progenitor cells predominantly repopulates the B-1 and not the conventional B-2 (CD5-) lineage (88, 89). Given our current understanding, it seems plausible that CD5+ and ABCs represent distinct B cell lineages that have converged on a similar role within the host immune response and corollary potential to recognize tumors. In this sense, while ABCs might participate in follicular or extrafollicular clonal redemption, CD5+ B cells would seem to only participate in extrafollicular clonal redemption in cancer (**Figure 1**).

## Role in paraneoplastic syndromes, autoimmunity, and checkpoint inhibition

Autoimmune paraneoplastic syndromes, though a relatively rare occurrence, have been well described clinically. These include organ specific effects such as pemphigus in CLL and retinopathy in breast cancer, or systemic autoimmune symptoms such as SLE in non-small cell lung cancer and cholangiocarcinoma or systemic sclerosis in breast and lung cancer (90). Many of these syndromes have been associated with biologically active autoantibodies; however, there are distinctions in the molecular underpinnings between paraneoplastic syndromes and their non-malignant autoimmune counterparts despite clinically similar presentations. For example, in paraneoplastic pemphigus, isolated autoantibodies recognized more distinct autoantigens, different epitopes on the same autoantigen, and were primarily of subclass IgG1 and IgG2 when compared to pemphigus vulgaris which had primarily IgG4 autoantibodies (91). A combination of autoantibodies to tumor associated antigens as well as autoantibodies to paraneoplastic syndrome associated antigens have been investigated as biomarkers in lung cancer and ovarian cancer (92, 93). Despite this, there has been little direct evidence for linkage of the same self-antigen expression by the tumor and the target autoimmune tissue. Many paraneoplastic markers are autoantibodies to intracellular proteins and are present in both paraneoplastic syndromes, and underlying tumors (94). This, combined with the evidence of epitope spreading in paraneoplastic syndromes suggests the loss of peripheral tolerance in cancer is complex and may be distinct from the processes that result in other well defined autoimmune disease.

Immune checkpoint inhibition (ICI) may present an opportunity to better understand the mechanisms underlying this loss of tolerance. Anti PD-1 and CTLA-4 treatment has resulted in distant organ-specific autoimmune adverse events, even in cancer types that had not previously been associated with spontaneously occurring paraneoplastic syndromes (95). As these adverse events may be more agent specific rather than specific to the underlying tumor, it could suggest activation of preexisting autoreactive T and B cells. Mechanisms proposed include release of inhibition by regulatory T cells resulting in greater T:B cell cross talk, and direct activation of Tbet+ B cells (96). Importantly, “CD21lo” B cells are increased in patients with melanoma who are treated with anti-CTLA4 or anti-PD1 plus anti-CTLA4, and these cells were transcriptionally suggestive of ABCs. Similarly, patients treated with a combination of anti-PD1 and anti-CTLA4 are more likely to develop immune related adverse events (97).

Autoimmunity in cancer has been demonstrated to have positive prognostic value in a number of settings. Patients who develop immune adverse events following ICI have improved response to treatment (98–101) and patients who develop paraneoplastic syndromes or spontaneous autoimmunity have improved outcomes (102, 103). In patients who had a preexisting autoimmune condition affecting an organ that subsequently developed cancer, the tumors were small and less invasive, such as in thyroid cancer arising in the background of thyroiditis (104). Hence, a breakdown in tolerance may provide a beneficial anti-tumor response, but successful clonal redemption may mitigate the undesirable autoimmune effects while focusing the response on tumor-specific antigens. Identification of mutated autoreactive clones might act as a prognostic biomarker and evidence for clonal redemption, though this has yet to be accomplished.

## Prognostic and therapeutic potential

ABCs may participate in promoting autoimmunity by presenting self-antigens to potentially autoreactive T cells. Indeed, in mouse spleens these cells colocalize with T cells at the T:B cell border, express MHCII and costimulatory molecules (CD80, CD86), and potently induce T cell proliferation (105). This suggests these cells, or their clonally redeemed analogues, may be able to effectively stimulate tumor-specific T cell responses to overexpressed self or near-self tumor antigens. For example, using a murine model of lymphoma, intratumoral injection with an IL-12-Fc fusion protein and TLR9 agonist (CpG) can lead to elimination of the injected primary tumor as well as a secondary tumor site. Depletion of B cells using anti-CD20 antibodies abrogated this effect, which was also contingent upon the presence of T cells (106). Thus, vaccination strategies aimed at intratumoral B cells can drive improved T cell responses in both primary and distant tumor sites.

Despite their state of hyporesponsiveness, ABCs can also be activated to differentiate into ASCs, supplying antibodies targeting tumor associated antigens. Tbet-mediated CSR preferentially drives production of IgG1 and IgG3 isotypes (107) and these antibodies are anticipated to effectively incite tumor cell killing through either complement dependent cytotoxicity or antibody dependent cellular cytotoxicity (108). In this sense, immunization of mice and rhesus macaques with HIV-1 envelope protein (which exhibits molecular mimicry with host kynureninase) and a TLR4 ligand with or without alum can stimulate anergic B cells to undergo differentiation into ASCs without overt autoimmunity (109). It is encouraging to speculate that this reflects an ability to therapeutically target ABCs. Indeed, targeting ABCs with vaccine strategies may be a convenient mechanism to instigate production of tumor-specific antibodies, as prior work has demonstrated that ABCs are poised to form GCs (47, 110). Moreover, the presence of intratumoral GC and TLS tend to predict response to ICI. Consistent with this, using multiple models of murine TNBC with increased tumor mutational burden, Hollern et al. demonstrated that response to anti-PD1/anti-CTLA4 was critically dependent upon CD4 T cells, B cells, IL-21, and secreted antibodies. The dominant intratumoral CD4 T cell transcriptional signature corresponded with that of T follicular helper cells (Tfh) and, alongside the requirement for IL-21, suggested that Tfh:B cell interaction drives response to dual ICI. They additionally showed increased accumulation of CD19+, CD80+, CD86+, MHCII+ B cells and production of IgG1 and IgG3 following dual ICI, which could suggest participation of ABCs in this mechanism. Efficacy of dual ICI was lost when antibody secretion was impaired or blocked (111). These results collectively suggest a framework wherein ABCs might be therapeutically targeted to produce tumor-specific antibodies—potentially through TLS formation and clonal redemption—while also stimulating tumor-specific T cell responses. Continued investigations into the specificity of ABCs and functionality within the TME will be required to determine the clinical utility of such an approach.

## Conclusions

In this review we have discussed the phenomenon of clonal redemption of B cells and how it may participate in the anti-tumor immune response. Tumor antigens include neoantigens, over or ectopically expressed native proteins, altered post-translational modifications, or alterations of protein structure within the TME. ABCs might then recognize these antigens, become activated, and produce tumor reactive antibodies, representing clonal redemption in either a GC dependent or independent manner. A similar mechanism of extrafollicular clonal redemption may exist for B1 cells. Interestingly, cancer patients frequently harbor tumor-reactive antibodies but do not incur systemic autoimmunity. The reasons for this remain to be elucidated but may represent a local breach of tolerance within the tumor and/or draining lymph node but not systemically. Nonetheless, further investigation is merited as clonal redemption of anergic B cell populations could provide a potent substrate with which to amplify immunotherapeutic modalities.

## Data availability statement

The original contributions presented in the study are included in the article/supplementary material. Further inquiries can be directed to the corresponding author.

## Author contributions

TM: Conceptualization, Writing – original draft. SL: Writing – original draft. JC: Conceptualization, Writing – review & editing.

## References

[1] Wardemann H Yurasov S Schaefer A Young JW Meffre E Nussenzweig MC. Predominant autoantibody production by early human b cell precursors. Science (2003) 301(5638):1374–7. doi: 10.1126/science.1086907 12920303

[2] Burnett DL Langley DB Schofield P Hermes JR Chan TD Jackson J. Germinal center antibody mutation trajectories are determined by rapid self/foreign discrimination. Science (2018) 360(6385):223–6. doi: 10.1126/science.aao3859 PMC5922412 29650674

[3] Lerner RA. Rare antibodies from combinatorial libraries suggests an S.O.S. component of the human immunological repertoire. Mol Biosyst (2011) 7(4):1004–12. doi: 10.1039/C0MB00310G 21298133

[4] Sabouri Z Schofield P Horikawa K Spierings E Kipling D Randall KL. Redemption of autoantibodies on anergic b cells by variable-region glycosylation and mutation away from self-reactivity. Proc Natl Acad Sci (2014) 111(25):E2567–E75. doi: 10.1073/pnas.1406974111 PMC4078846 24821781

[5] Reed JH Jackson J Christ D Goodnow CC. Clonal redemption of autoantibodies by somatic hypermutation away from self-reactivity during human immunization. J Exp Med (2016) 213(7):1255–65. doi: 10.1084/jem.20151978 PMC4925023 27298445

[6] Lu H Lou H Wengert G Paudel R Patel N Desai S. Tumor and local lymphoid tissue interaction determines prognosis in high-grade serous ovarian cancer. Cell Rep Med (2023) 4(7):101092. doi: 10.1016/j.xcrm.2023.101092 37348499 PMC10394173

[7] Berntsson J Nodin B Eberhard J Micke P Jirström K. Prognostic impact of tumour-infiltrating b cells and plasma cells in colorectal cancer. Int J Cancer (2016) 139(5):1129–39. doi: 10.1002/ijc.30138 27074317

[8] Hennequin A Derangère V Boidot R Apetoh L Vincent J Orry D. Tumor infiltration by tbet+ effector t cells and CD20+ b cells is associated with survival in gastric cancer patients. Oncoimmunology (2016) 5(2):e1054598. doi: 10.1080/2162402X.2015.1054598 27057426 PMC4801425

[9] Willsmore ZN Harris RJ Crescioli S Hussein K Kakkassery H Thapa D. B cells in patients with melanoma: Implications for treatment with checkpoint inhibitor antibodies. Front Immunol (2021) 11:622442. doi: 10.3389/fimmu.2020.622442 33569063 PMC7868381

[10] Petitprez F de Reyniès A Keung EZ Chen TW Sun CM Calderaro J. B cells are associated with survival and immunotherapy response in sarcoma. Nature (2020) 577(7791):556–60. doi: 10.1038/s41586-019-1906-8 31942077

[11] Lao XM Liang YJ Su YX Zhang SE Zhou XI Liao GQ. Distribution and significance of interstitial fibrosis and stroma-infiltrating b cells in tongue squamous cell carcinoma. Oncol Lett (2016) 11(3):2027–34. doi: 10.3892/ol.2016.4184 PMC4774478 26998116

[12] Nedergaard BS Ladekarl M Nyengaard JR Nielsen K. A comparative study of the cellular immune response in patients with stage IB cervical squamous cell carcinoma. low numbers of several immune cell subtypes are strongly associated with relapse of disease within 5 years. Gynecologic Oncol (2008) 108(1):106–11. doi: 10.1016/j.ygyno.2007.08.089 17945335

[13] Wang S-S Liu W Ly D Xu H Qu L Zhang L. Tumor-infiltrating b cells: their role and application in anti-tumor immunity in lung cancer. Cell Mol Immunol (2019) 16(1):6–18. doi: 10.1038/s41423-018-0027-x 29628498 PMC6318290

[14] Buisseret L Garaud S de Wind A Van den Eynden G Boisson A Solinas C. Tumor-infiltrating lymphocyte composition, organization and PD-1/ PD-L1 expression are linked in breast cancer. Oncoimmunology (2017) 6(1):e1257452. doi: 10.1080/2162402X.2016.1257452 28197375 PMC5283629

[15] Garaud S Buisseret L Solinas C Gu-Trantien C de Wind A Van den Eynden G. Tumor-infiltrating b cells signal functional humoral immune responses in breast cancer. JCI Insight (2019) 4(18):1–20. doi: 10.1172/jci.insight.129641 PMC6795287 31408436

[16] Yang R Han Y Yi W Long Q. Autoantibodies as biomarkers for breast cancer diagnosis and prognosis. Front Immunol (2022) 13. doi: 10.3389/fimmu.2022.1035402 PMC9701846 36451832

[17] Niloofa R De Zoysa MI Seneviratne SL. Autoantibodies in the diagnosis, prognosis, and prediction of colorectal cancer. J Cancer Res Ther (2021) 17(4):819–33. doi: 10.4103/jcrt.JCRT_64_19 34528528

[18] Xu Y Zhang W Xia T Liu Y Bi Z Guo L. Diagnostic value of tumor-associated autoantibodies panel in combination with traditional tumor markers for lung cancer. Front Oncol (2023) 13:1022331. doi: 10.3389/fonc.2023.1022331 36874112 PMC9975551

[19] Zhang X Liu M Zhang X Wang Y Dai L. Chapter one - autoantibodies to tumor-associated antigens in lung cancer diagnosis. In: Makowski GS, editor. Advances in clinical chemistry. (New York, NY, USA: Elsevier) (2021) 103:1–45. 10.1016/bs.acc.2020.08.005 34229848

[20] Laumont CM Banville AC Gilardi M Hollern DP Nelson BH. Tumour-infiltrating b cells: immunological mechanisms, clinical impact and therapeutic opportunities. Nat Rev Cancer (2022) 22(7):414–30. doi: 10.1038/s41568-022-00466-1 PMC9678336 35393541

[21] Moir S Ho J Malaspina A Wang W DiPoto AC O'Shea MA. Evidence for HIV-associated b cell exhaustion in a dysfunctional memory b cell compartment in HIV-infected viremic individuals. J Exp Med (2008) 205(8):1797–805. doi: 10.1084/jem.20072683 PMC2525604 18625747

[22] Li H Borrego F Nagata S Tolnay M. Fc receptor–like 5 expression distinguishes two distinct subsets of human circulating tissue–like memory b cells. J Immunol (2016) 196(10):4064–74. doi: 10.4049/jimmunol.1501027 27076679

[23] Weiss GE Crompton PD Li S Walsh LA Moir S Traore B. Atypical memory b cells are greatly expanded in individuals living in a malaria-endemic area. J Immunol (2009) 183(3):2176–82. doi: 10.4049/jimmunol.0901297 PMC2713793 19592645

[24] Kim CC Baccarella AM Bayat A Pepper M Fontana MF. FCRL5(+) memory b cells exhibit robust recall responses. Cell Rep (2019) 27(5):1446–60.e4. doi: 10.1016/j.celrep.2019.04.019 31042472 PMC6530801

[25] Rubtsov AV Rubtsova K Fischer A Meehan RT Gillis JZ Kappler JW. Toll-like receptor 7 (TLR7)-driven accumulation of a novel CD11c⁺ b-cell population is important for the development of autoimmunity. Blood (2011) 118(5):1305–15. doi: 10.1182/blood-2011-01-331462 PMC3152497 21543762

[26] Naradikian MS Myles A Beiting DP Roberts KJ Dawson L Herati RS. Cutting edge: IL-4, IL-21, and IFN-γ interact to govern t-bet and CD11c expression in TLR-activated b cells. J Immunol (2016) 197(4):1023–8. doi: 10.4049/jimmunol.1600522 PMC4975960 27430719

[27] Holla P Dizon B Ambegaonkar AA Rogel N Goldschmidt E Boddapati AK. Shared transcriptional profiles of atypical b cells suggest common drivers of expansion and function in malaria, HIV, and autoimmunity. Sci Adv (2021) 7(22):1–18. doi: 10.1126/sciadv.abg8384 PMC8153733 34039612

[28] Fontana MF Baccarella A Craft JF Boyle MJ McIntyre TI Wood MD. A novel model of asymptomatic plasmodium parasitemia that recapitulates elements of the human immune response to chronic infection. PloS One (2016) 11(9):e0162132. doi: 10.1371/journal.pone.0162132 27583554 PMC5008831

[29] Becker AM Dao KH Han BK Kornu R Lakhanpal S Mobley AB. SLE peripheral blood b cell, t cell and myeloid cell transcriptomes display unique profiles and each subset contributes to the interferon signature. PloS One (2013) 8(6):e67003. doi: 10.1371/journal.pone.0067003 23826184 PMC3691135

[30] Wang S Wang J Kumar V Karnell JL Naiman B Gross PS. IL-21 drives expansion and plasma cell differentiation of autoreactive CD11chiT-bet+ b cells in SLE. Nat Commun (2018) 9(1):1758. doi: 10.1038/s41467-018-03750-7 29717110 PMC5931508

[31] Muellenbeck MF Ueberheide B Amulic B Epp A Fenyo D Busse CE. Atypical and classical memory b cells produce plasmodium falciparum neutralizing antibodies. J Exp Med (2013) 210(2):389–99. doi: 10.1084/jem.20121970 PMC3570107 23319701

[32] Hopp CS Sekar P Diouf A Miura K Boswell K Skinner J. Plasmodium falciparum-specific IgM b cells dominate in children, expand with malaria, and produce functional IgM. J Exp Med (2021) 218(4):1–15. doi: 10.1084/jem.20200901 PMC7938365 33661303

[33] Kochayoo P Thawornpan P Wangriatisak K Changrob S Leepiyasakulchai C Khowawisetsut L. Interferon-γ signal drives differentiation of t-bethi atypical memory b cells into plasma cells following plasmodium vivax infection. Sci Rep (2022) 12(1):4842. doi: 10.1038/s41598-022-08976-6 35318412 PMC8941117

[34] Yu B Qi Y Li R Shi Q Satpathy AT Chang HY. B cell-specific XIST complex enforces x-inactivation and restrains atypical b cells. Cell (2021) 184(7):1790–803.e17. doi: 10.1016/j.cell.2021.02.015 33735607 PMC9196326

[35] Norouzian M Mehdipour F Balouchi Anaraki S Ashraf MJ Khademi B Ghaderi A. Atypical memory and regulatory b cell subsets in tumor draining lymph nodes of head and neck squamous cell carcinoma correlate with good prognostic factors. Head Neck Pathol (2020) 14(3):645–56. doi: 10.1007/s12105-019-01095-1 PMC7413970 31691165

[36] Nielsen JS Sahota RA Milne K Kost SE Nesslinger NJ Watson PH. CD20+ tumor-infiltrating lymphocytes have an atypical CD27– memory phenotype and together with CD8+ t cells promote favorable prognosis in ovarian cancer. Clin Cancer Res (2012) 18(12):3281–92. doi: 10.1158/1078-0432.CCR-12-0234 22553348

[37] Shi J-Y Gao Q Wang Z-C Zhou J Wang X-Y Min Z-H. Margin-infiltrating CD20+ b cells display an atypical memory phenotype and correlate with favorable prognosis in hepatocellular carcinoma. Clin Cancer Res (2013) 19(21):5994–6005. doi: 10.1158/1078-0432.CCR-12-3497 24056784

[38] Carpenter E Alaguthurai T Hossain F Graham R Kakkassery H Keane S. Abstract P2-20-02: Enrichment of atypical memory double negative (CD27— IgD—) tumour infiltrating b cells following neoadjuvant chemotherapy for early-stage breast cancer. Cancer Res (2023) 83(5_Supplement):P2–20-02-P2-20-02. doi: 10.1158/1538-7445.SABCS22-P2-20-02

[39] Hao Y O'Neill P Naradikian MS Scholz JL Cancro MP. A b-cell subset uniquely responsive to innate stimuli accumulates in aged mice. Blood (2011) 118(5):1294–304. doi: 10.1182/blood-2011-01-330530 PMC3152496 21562046

[40] Rubtsova K Rubtsov AV van Dyk LF Kappler JW Marrack P. T-box transcription factor t-bet, a key player in a unique type of b-cell activation essential for effective viral clearance. Proc Natl Acad Sci U S A (2013) 110(34):E3216–24. doi: 10.1073/pnas.1312348110 PMC3752276 23922396

[41] Markine-Goriaynoff D Coutelier J-P. Increased efficacy of the immunoglobulin G2a subclass in antibody-mediated protection against lactate dehydrogenase-elevating virus-induced polioencephalomyelitis revealed with switch mutants. J Virology (2002) 76(1):432–5. doi: 10.1128/JVI.76.1.432-435.2002 PMC135718 11739710

[42] Coutelier JP van der Logt JT Heessen FW Vink A van Snick J. Virally induced modulation of murine IgG antibody subclasses. J Exp Med (1988) 168(6):2373–8. doi: 10.1084/jem.168.6.2373 PMC2189165 3199074

[43] Peng SL Szabo SJ Glimcher LH. T-bet regulates IgG class switching and pathogenic autoantibody production. Proc Natl Acad Sci (2002) 99(8):5545–50. doi: 10.1073/pnas.082114899 PMC122806 11960012

[44] Gerth AJ Lin L Peng SL. T-bet regulates t-independent IgG2a class switching. Int Immunol (2003) 15(8):937–44. doi: 10.1093/intimm/dxg093 12882831

[45] Pone EJ Zhang J Mai T White CA Li G Sakakura JK. BCR-signalling synergizes with TLR-signalling for induction of AID and immunoglobulin class-switching through the non-canonical NF-κB pathway. Nat Commun (2012) 3(1):767. doi: 10.1038/ncomms1769 22473011 PMC3337981

[46] Du SW Arkatkar T Jacobs HM Rawlings DJ Jackson SW. Generation of functional murine CD11c(+) age-associated b cells in the absence of b cell t-bet expression. Eur J Immunol (2019) 49(1):170–8. doi: 10.1002/eji.201847641 PMC6779321 30353919

[47] Rubtsova K Rubtsov AV Thurman JM Mennona JM Kappler JW Marrack P. B cells expressing the transcription factor t-bet drive lupus-like autoimmunity. J Clin Invest (2017) 127(4):1392–404. doi: 10.1172/JCI91250 PMC5373868 28240602

[48] Tian M Hua Z Hong S Zhang Z Liu C Lin L. B cell-intrinsic MyD88 signaling promotes initial cell proliferation and differentiation to enhance the germinal center response to a virus-like particle. J Immunol (2018) 200(3):937–48. doi: 10.4049/jimmunol.1701067 29282308

[49] Jackson SW Scharping NE Kolhatkar NS Khim S Schwartz MA Li QZ. Opposing impact of b cell-intrinsic TLR7 and TLR9 signals on autoantibody repertoire and systemic inflammation. J Immunol (2014) 192(10):4525–32. doi: 10.4049/jimmunol.1400098 PMC4041708 24711620

[50] Ehlers M Fukuyama H McGaha TL Aderem A Ravetch JV. TLR9/MyD88 signaling is required for class switching to pathogenic IgG2a and 2b autoantibodies in SLE. J Exp Med (2006) 203(3):553–61. doi: 10.1084/jem.20052438 PMC2118244 16492804

[51] Nguyen HV Mouly E Chemin K Luinaud R Despres R Fermand JP. The ets-1 transcription factor is required for Stat1-mediated t-bet expression and IgG2a class switching in mouse b cells. Blood (2012) 119(18):4174–81. doi: 10.1182/blood-2011-09-378182 22438254

[52] Kenderes KJ Levack RC Papillion AM Cabrera-Martinez B Dishaw LM Winslow GM. T-bet(+) IgM memory cells generate multi-lineage effector b cells. Cell Rep (2018) 24(4):824–37.e3. doi: 10.1016/j.celrep.2018.06.074 30044980 PMC6141031

[53] Rolink AG Andersson J Melchers F. Characterization of immature b cells by a novel monoclonal antibody, by turnover and by mitogen reactivity. Eur J Immunol (1998) 28(11):3738–48. doi: 10.1002/(SICI)1521-4141(199811)28:11<3738::AID-IMMU3738>3.0.CO;2-Q 9842916

[54] McDonald G Medina CO Pilichowska M Kearney JF Shinkura R Selsing E. Accelerated systemic autoimmunity in the absence of somatic hypermutation in 564Igi: A mouse model of systemic lupus with knocked-in heavy and light chain genes. Front Immunol (2017) 8:1094. doi: 10.3389/fimmu.2017.01094 28955333 PMC5601273

[55] Descatoire M Fritzen R Rotman S Kuntzelman G Leber XC Droz-Georget S. Critical role of WASp in germinal center tolerance through regulation of b cell apoptosis and diversification. Cell Rep (2022) 38(10):110474. doi: 10.1016/j.celrep.2022.110474 35263577

[56] Mazor RD Nathan N Gilboa A Stoler-Barak L Moss L Solomonov I. Tumor-reactive antibodies evolve from non-binding and autoreactive precursors. Cell (2022) 185(7):1208–22.e21. doi: 10.1016/j.cell.2022.02.012 35305314

[57] Stone B Schummer M Paley PJ Thompson L Stewart J Ford M. Serologic analysis of ovarian tumor antigens reveals a bias toward antigens encoded on 17q. Int J Cancer (2003) 104(1):73–84. doi: 10.1002/ijc.10900 12532422

[58] Gnjatic S Ritter E Büchler MW Giese NA Brors B Frei C. Seromic profiling of ovarian and pancreatic cancer. Proc Natl Acad Sci (2010) 107(11):5088–93. doi: 10.1073/pnas.0914213107 PMC2841879 20194765

[59] Cheever MA Allison JP Ferris AS Finn OJ Hastings BM Hecht TT. The prioritization of cancer antigens: a national cancer institute pilot project for the acceleration of translational research. Clin Cancer Res (2009) 15(17):5323–37. doi: 10.1158/1078-0432.CCR-09-0737 PMC5779623 19723653

[60] Laumont CM Nelson BH. B cells in the tumor microenvironment: Multi-faceted organizers, regulators, and effectors of anti-tumor immunity. Cancer Cell (2023) 41(3):466–89. doi: 10.1016/j.ccell.2023.02.017 36917951

[61] Prabhakar BS Saegusa J Onodera T Notkins AL. Lymphocytes capable of making monoclonal autoantibodies that react with multiple organs are a common feature of the normal b cell repertoire. J Immunol (1984) 133(6):2815–7. doi: 10.4049/jimmunol.133.6.2815 6333448

[62] Casali P Notkins AL. CD5+ b lymphocytes, polyreactive antibodies and the human b-cell repertoire. Immunol Today (1989) 10(11):364–8. doi: 10.1016/0167-5699(89)90268-5 2482031

[63] Burastero SE Casali P. Characterization of human CD5 (Leu-1, OKT1)+ b lymphocytes and the antibodies they produce. Contrib Microbiol Immunol (1989) 11:231–62. 2479500

[64] Dighiero G Guilbert B Fermand J-P Lymberi P Danon F Avrameas S. Thirty-six human monoclonal immunoglobulins with antibody activity against cytoskeleton proteins, thyroglobulin, and native DNA: Immunologic studies and clinical correlations. Blood (1983) 62(2):264–70. doi: 10.1182/blood.V62.2.264.264 6409187

[65] Ueki Y Goldfarb IS Harindranath N Gore M Koprowski H Notkins AL. Clonal analysis of a human antibody response. quantitation of precursors of antibody-producing cells and generation and characterization of monoclonal IgM, IgG, and IgA to rabies virus. J Exp Med (1990) 171(1):19–34. doi: 10.1084/jem.171.1.19 2153188 PMC2187652

[66] Burastero SE Casali P Wilder RL Notkins AL. Monoreactive high affinity and polyreactive low affinity rheumatoid factors are produced by CD5+ b cells from patients with rheumatoid arthritis. J Exp Med (1988) 168(6):1979–92. doi: 10.1084/jem.168.6.1979 PMC2189135 3264319

[67] Elkon K Casali P. Nature and functions of autoantibodies. Nat Clin Pract Rheumatol (2008) 4(9):491–8. doi: 10.1038/ncprheum0895 PMC2703183 18756274

[68] Notkins AL. Polyreactivity of antibody molecules. Trends Immunol (2004) 25(4):174–9. doi: 10.1016/j.it.2004.02.004 15039043

[69] Sanz I Casali P Thomas J Notkins A Capra J. Genetic basis of natural autoantibodies: organization, complexity and mechanisms of diversity of the human b cell repertoire. J Immunol (1989) 11:4054–61. doi: 10.4049/jimmunol.142.11.4054 2497188

[70] Schettino EW Chai SK Kasaian MT Schroeder HW Jr. Casali P. VHDJH gene sequences and antigen reactivity of monoclonal antibodies produced by human b-1 cells: evidence for somatic selection. J Immunol (1997) 158(5):2477–89. doi: 10.4049/jimmunol.158.5.2477 PMC4631314 9037000

[71] Mageed RA MacKenzie LE Stevenson FK Yuksel B Shokri F Maziak BR. Selective expression of a VHIV subfamily of immunoglobulin genes in human CD5+ b lymphocytes from cord blood. J Exp Med (1991) 174(1):109–13. doi: 10.1084/jem.174.1.109 PMC2118889 1711557

[72] Grillot-Courvalin C Brouet JC Piller F Rassenti LZ Labaume S Silverman GJ. An anti-b cell autoantibody from wiskott-aldrich syndrome which recognizes i blood group specificity on normal human b cells. Eur J Immunol (1992) 22(7):1781–8. doi: 10.1002/eji.1830220717 1623923

[73] Schickel JN Glauzy S Ng YS Chamberlain N Massad C Isnardi I. Self-reactive VH4-34-expressing IgG b cells recognize commensal bacteria. J Exp Med (2017) 214(7):1991–2003. doi: 10.1084/jem.20160201 28500047 PMC5502416

[74] Avrameas S. Natural autoantibodies: from 'horror autotoxicus' to 'gnothi seauton'. Immunol Today (1991) 12(5):154–9. doi: 10.1016/s0167-5699(05)80045-3 1715166

[75] Pugh-Bernard AE Silverman GJ Cappione AJ Villano ME Ryan DH Insel RA. Regulation of inherently autoreactive VH4-34 b cells in the maintenance of human b cell tolerance. J Clin Invest (2001) 108(7):1061–70. doi: 10.1172/JCI200112462 PMC200949 11581307

[76] Cappione A 3rd Anolik JH Pugh-Bernard A Barnard J Dutcher P Silverman G. Germinal center exclusion of autoreactive b cells is defective in human systemic lupus erythematosus. J Clin Invest (2005) 115(11):3205–16. doi: 10.1172/JCI24179 PMC1242189 16211091

[77] Thomas D Rathinavel AK Radhakrishnan P. Altered glycosylation in cancer: A promising target for biomarkers and therapeutics. Biochim Biophys Acta (BBA) - Rev Cancer (2021) 1875(1):188464. doi: 10.1016/j.bbcan.2020.188464 PMC7855613 33157161

[78] Brändlein S Pohle T Ruoff N Wozniak E Müller-Hermelink HK Vollmers HP. Natural IgM antibodies and immunosurveillance mechanisms against epithelial cancer cells in humans. Cancer Res (2003) 63(22):7995–8005. 14633732

[79] Thomas MD Clough K Melamed MD Stevenson FK Chapman CJ Spellerberg MB. A human monoclonal antibody encoded by the v 4-34 gene segment recognises melanoma-associated ganglioside *via* CDR3 and FWR1. Hum Antibodies (1999) 9:95–106. doi: 10.3233/HAB-1999-9203 10405830

[80] Brändlein S Lorenz J Ruoff N Hensel F Beyer I Müller J. Human monoclonal IgM antibodies with apoptotic activity isolated from cancer patients. Hum Antibodies (2002) 11(4):107–19. doi: 10.3233/HAB-2002-11401 12775891

[81] Brändlein S Eck M Ströbel P Wozniak E Müller-Hermelink HK Hensel F. PAM-1, a natural human IgM antibody as new tool for detection of breast and prostate precursors. Hum Antibodies (2004) 13:97–104. doi: 10.3233/HAB-2004-13401 15719499

[82] Mohan C Morel L Yang P Wakeland EK. Accumulation of splenic B1a cells with potent antigen-presenting capability in NZM2410 lupus-prone mice. Arthritis Rheumatol (1998) 41(9):1652–62. doi: 10.1002/1529-0131(199809)41:9<1652::AID-ART17>3.0.CO;2-W 9751099

[83] Hippen KL Tze LE Behrens TW. Cd5 maintains tolerance in anergic b cells. J Exp Med (2000) 191(5):883–90. doi: 10.1084/jem.191.5.883 PMC2195862 10704468

[84] Masle-Farquhar E Peters TJ Miosge LA Parish IA Weigel C Oakes CC. Uncontrolled CD21low age-associated and B1 b cell accumulation caused by failure of an EGR2/3 tolerance checkpoint. Cell Rep (2022) 38(3):110259. doi: 10.1016/j.celrep.2021.110259 35045301

[85] Kasaian MT Ikematsu H Casali P. Identification and analysis of a novel human surface CD5- b lymphocyte subset producing natural antibodies. J Immunol (1992) 148(9):2690–702. doi: 10.4049/jimmunol.148.9.2690 PMC4626883 1374094

[86] Harris DP Goodrich S Gerth AJ Peng SL Lund FE. Regulation of IFN-γ production by b effector 1 cells: Essential roles for t-bet and the IFN-γ Receptor1. J Immunol (2005) 174(11):6781–90. doi: 10.4049/jimmunol.174.11.6781 15905519

[87] Ishida H Hastings R Kearney J Howard M. Continuous anti-interleukin 10 antibody administration depletes mice of ly-1 b cells but not conventional b cells. J Exp Med (1992) 175(5):1213–20. doi: 10.1084/jem.175.5.1213 PMC2119200 1533240

[88] Solvason N Lehuen A Kearney JF. An embryonic source of Ly1 but not conventional b cells. Int Immunol (1991) 3(6):543–50. doi: 10.1093/intimm/3.6.543 1679664

[89] Kantor AB Stall AM Adams S Herzenberg LA Herzenberg LA. Differential development of progenitor activity for three b-cell lineages. Proc Natl Acad Sci USA (1992) 89(8):3320–4. doi: 10.1073/pnas.89.8.3320 PMC48858 1565622

[90] Maverakis E Goodarzi H Wehrli LN Ono Y Garcia MS. The etiology of paraneoplastic autoimmunity. Clin Rev Allergy Immunol (2012) 42(2):135–44. doi: 10.1007/s12016-010-8248-5 21246308

[91] Futei Y Amagai M Hashimoto T Nishikawa T. Conformational epitope mapping and IgG subclass distribution of desmoglein 3 in paraneoplastic pemphigus. J Am Acad Dermatol (2003) 49(6):1023–8. doi: 10.1016/S0190-9622(03)02160-1 14639380

[92] Maddison P Titulaer MJ Verschuuren JJ Gozzard P Lang B Irani SR. The utility of anti-SOX2 antibodies for cancer prediction in patients with paraneoplastic neurological disorders. J Neuroimmunol (2019) 326:14–8. doi: 10.1016/j.jneuroim.2018.11.003 PMC6375907 30445363

[93] Hurley LC Levin NK Chatterjee M Coles J Muszkat S Howarth Z. Evaluation of paraneoplastic antigens reveals TRIM21 autoantibodies as biomarker for early detection of ovarian cancer in combination with autoantibodies to NY-ESO-1 and TP53. Cancer biomark (2020) 27(3):407–21. doi: 10.3233/CBM-190988 PMC8076913 32083570

[94] Pittock SJ Kryzer TJ Lennon VA. Paraneoplastic antibodies coexist and predict cancer, not neurological syndrome. Ann Neurol (2004) 56(5):715–9. doi: 10.1002/ana.20269 15468074

[95] Ramos-Casals M Brahmer JR Callahan MK Flores-Chávez A Keegan N Khamashta MA. Immune-related adverse events of checkpoint inhibitors. Nat Rev Dis Primers (2020) 6(1):38. doi: 10.1038/s41572-020-0160-6 32382051 PMC9728094

[96] Dhodapkar KM Duffy A Dhodapkar MV. Role of b cells in immune-related adverse events following checkpoint blockade. Immunol Rev (2023) 318:89–95. doi: 10.1111/imr.13238 PMC10530150 37421187

[97] Das R Bar N Ferreira M Newman AM Zhang L Bailur JK. Early b cell changes predict autoimmunity following combination immune checkpoint blockade. J Clin Invest (2018) 128(2):715–20. doi: 10.1172/JCI96798 PMC5785243 29309048

[98] Riudavets M Barba A Maroto P SULLIVAN IG Anguera G Páez D. Correlation between immune-related adverse events (irAEs) and efficacy in patients with solid tumors treated with immune-checkpoints inhibitors (ICIs). J Clin Oncol (2018) 36(15_suppl):3064–4. doi: 10.1200/JCO.2018.36.15_suppl.3064

[99] Okada N Kawazoe H Takechi K Matsudate Y Utsunomiya R Zamami Y. Association between immune-related adverse events and clinical efficacy in patients with melanoma treated with nivolumab: A multicenter retrospective study. Clin Ther (2019) 41(1):59–67. doi: 10.1016/j.clinthera.2018.11.004 30528047

[100] Elias R Yan F Singla N Levonyack N Formella J Christie A. Immune-related adverse events are associated with improved outcomes in ICI-treated renal cell carcinoma patients. J Clin Oncol (2019) 37(7_suppl):645–5. doi: 10.1200/JCO.2019.37.7_suppl.645

[101] Sato K Akamatsu H Murakami E Sasaki S Kanai K Hayata A. Correlation between immune-related adverse events and efficacy in non-small cell lung cancer treated with nivolumab. Lung Cancer (2018) 115:71–4. doi: 10.1016/j.lungcan.2017.11.019 29290265

[102] Motofei IG. Melanoma and autoimmunity: spontaneous regressions as a possible model for new therapeutic approaches. Melanoma Res (2019) 29(3):231–6. doi: 10.1097/CMR.0000000000000573 30615013

[103] Maddison P Gozzard P Grainge MJ Lang B. Long-term survival in paraneoplastic lambert-eaton myasthenic syndrome. Neurology (2017) 88(14):1334–9. doi: 10.1212/WNL.0000000000003794 28251917

[104] Moon S Chung HS Yu JM Yoo HJ Park JH Kim DS. Associations between hashimoto thyroiditis and clinical outcomes of papillary thyroid cancer: A meta-analysis of observational studies. Endocrinol Metab (Seoul) (2018) 33(4):473–84. doi: 10.3803/EnM.2018.33.4.473 PMC6279904 30513562

[105] Rubtsov AV Rubtsova K Kappler JW Jacobelli J Friedman RS Marrack P. CD11c-expressing b cells are located at the t Cell/B cell border in spleen and are potent APCs. J Immunol (2015) 195(1):71–9. doi: 10.4049/jimmunol.1500055 PMC4475418 26034175

[106] Sagiv Barfi I Czerwinski DK Levy R. *In situ* vaccination with IL-12Fc and TLR agonist - a crucial role for b cells in generating anti-tumor t cell immunity. Blood (2021) 138:3514. doi: 10.1182/blood-2021-146628

[107] Knox JJ Buggert M Kardava L Seaton KE Eller MA Canaday DH. T-bet+ b cells are induced by human viral infections and dominate the HIV gp140 response. JCI Insight (2017) 2(8):1–16. doi: 10.1172/jci.insight.92943 PMC5396521 28422752

[108] Brüggemann M Williams GT Bindon CI Clark MR Walker MR Jefferis R. Comparison of the effector functions of human immunoglobulins using a matched set of chimeric antibodies. J Exp Med (1987) 166(5):1351–61. doi: 10.1084/jem.166.5.1351 PMC2189658 3500259

[109] Bradley T Yang G Ilkayeva O Holl TM Zhang R Zhang J. HIV-1 envelope mimicry of host enzyme kynureninase does not disrupt tryptophan metabolism. J Immunol (2016) 197(12):4663–73. doi: 10.4049/jimmunol.1601484 PMC5136304 27849170

[110] Domeier PP Chodisetti SB Soni C Schell SL Elias MJ Wong EB. IFN-γ receptor and STAT1 signaling in b cells are central to spontaneous germinal center formation and autoimmunity. J Exp Med (2016) 213(5):715–32. doi: 10.1084/jem.20151722 PMC4854731 27069112

[111] Hollern DP Xu N Thennavan A Glodowski C Garcia-Recio S Mott KR. B cells and t follicular helper cells mediate response to checkpoint inhibitors in high mutation burden mouse models of breast cancer. Cell (2019) 179(5):1191–206. e21. doi: 10.1016/j.cell.2019.10.028 31730857 PMC6911685

